# Mechanical Thrombectomy in Acute Ischemic Stroke COVID-19 and Non-COVID-19 Patients: A Single Comprehensive Stroke Center Study

**DOI:** 10.3390/life13010186

**Published:** 2023-01-09

**Authors:** Sanja Lovrić Kojundžić, Sara Sablić, Danijela Budimir Mršić, Maja Marinović Guić, Ivan Kraljević, Benjamin Benzon, Dragan Dragičević

**Affiliations:** 1 Department of Diagnostic and Interventional Radiology, University Hospital of Split, 21000 Split, Croatia; 2 School of Medicine, University of Split, 21000 Split, Croatia; 3 University Department of Health Studies, University of Split, 21000 Split, Croatia

**Keywords:** COVID-19, mechanical thrombectomy, acute ischemic stroke, neutrophil/lymphocyte ratio

## Abstract

Coronavirus disease 2019 (COVID-19) increases the risk for thromboembolic events, such as acute ischemic stroke (AIS). Mechanical thrombectomy (MT) is a therapy of choice in early diagnosed AIS; however, its success and outcomes in COVID-19 patients are contradictory. This study presented our experience with MT performed in COVID-19 patients compared to a control group. The retrospective analysis included patients with AIS who underwent MT from April 2021 to April 2022 at our institution. There were 13 COVID-19-related patients (with active or past COVID-19 infection) and 55 non-COVID-19 patients (negative COVID-19 status). We analyzed patients’ baseline clinical and laboratory data, modified Thrombolysis in Cerebral Infarction (mTICI) scale, used 24 h follow-up CT findings, and modified the Rankin scale. The COVID-19 group had higher values of leukocytes, neutrophils, neutrophil/leukocyte ratios, ASL, ALT, LDH and CRP, and lower values of lymphocytes compared to the control group. The AIS mostly occurred in posterior circulation in the COVID-19 group, while anterior circulation was more affected in the control group. Treatment approach and successful reperfusion did not differ between groups. In conclusion, although differences in some clinical and laboratory parameters between COVID-19 and non-COVID-19 groups were found, the outcomes of mechanical thrombectomy were equal.

## 1. Introduction

Coronavirus disease (COVID-19) is an infectious disease caused by the SARS-CoV-2 virus. COVID-19 is not only a respiratory system infection, but a multisystem disease associated with a number of extrapulmonary complications. These include a variety of different involvements throughout multiple organ systems that manifest as thromboembolic complications, cardiac disorders, liver or kidney injuries, gastrointestinal symptoms, neurologic illnesses, ocular symptoms, and many others [1,2]. Thromboembolic events are common and significantly contribute to the increased morbidity and mortality of COVID-19 patients. There are several inflammatory and prothrombotic mechanisms considered to be responsible for thrombosis in COVID-19, such as endothelial dysfunction, exaggerated inflammatory response that induces “cytokine storm”, and hypercoagulable state [3,4]. Since the onset of the COVID-19 pandemic, a considerable percentage of infected patients had documented thrombotic complications, including acute ischemic stroke (AIS). The stroke incidence in COVID-19 patients was up to 1.4%, especially in those severely infected patients who had pre-existing cardiovascular risk factors [5,6].

Mechanical thrombectomy (MT) has been widely accepted as the first-line therapy for patients with emergent large vessel occlusion (ELVO) stroke [7,8], and has also been implemented in the management of ELVO stroke in COVID-19 patients. However, during the COVID-19 pandemic, many MT procedures were prolonged or even canceled, and there are conflicting results as to whether the pandemic influenced clinical outcomes and procedure-related serious adverse events [9,10]. The aim of our study was to present a single center experience on mechanical thrombectomy outcomes performed in COVID-19 and non-COVID-19 patients.

## 2. Materials and Methods

The study complied with the ethical guidelines of the 1975 Declaration of Helsinki, and the Institutional Ethics Committee approved it at the University hospital of Split, Croatia. Given the retrospective research setting, the informed consent form was waived. This was a retrospective study of patients with AIS who underwent mechanical thrombectomy for ELVO from April 2021 to April 2022 at University Hospital of Split. Patients who met inclusion criteria were divided into two groups based on their COVID-19 status: the COVID-19 and the non-COVID-19 group. The COVID-19 group consisted of patients with active or past COVID-19 infection (up to 12 months) and non-COVID-19 group was SARS-CoV-2 negative at admission with no reported COVID-19 infection in the past. We excluded patients who did not have complete laboratory tests and were lacking outcome information. Both groups of patients underwent endovascular treatment due to occlusion in any segment of cerebral arteries causing anterior or posterior stroke. Posterior circulation included basilar artery and posterior cerebral arteries while anterior circulation included anterior and middle cerebral artery territories and distal internal carotid artery. Before endovascular treatment, all patients underwent Stroke protocol consisting of an emergent head CT scan, cerebral CT angiography (CTA), and CT perfusion (CTP) to determine those who met the criteria for MT. Interventional neuroradiologists with expertise in acute stroke endovascular operations conducted MT, under local or general anesthesia. The endovascular procedure was based on standard thrombectomy techniques with approved endovascular devices using aspiration catheters or stent-retrievers, or a combination of both. Patients’ demographic (age, sex, smoking, stroke risk factors, comorbidity, anticoagulant therapy), laboratory and clinical data (i.e., location of stroke, MT technique used, modified Rankin Scale (mRS), National Institutes of Health Stroke Scale (NIHSS) on admission and discharge, and modified thrombolysis in cerebral infarction (mTICI) scale) were gathered from the hospital’s information system. The early postinterventional and later neurological outcomes (occlusion, symptomatic intracranial hemorrhage—sICH, in-hospital death) were also collected and analyzed. Complete reperfusion was marked as an mTICI scale score of 3, while successful reperfusion was defined as mTICI 2B, 2C and 3. The clinical efficacy outcome was the rate of functional independence measured by the mRS ranging from 0 to 6, with 0 meaning no symptoms/disability and 6 meaning death. A good mRS outcome was defined as 0–2 at discharge and 90 days after stroke. We also analyzed head CT scans of the patients 24 h after the mechanical thrombectomy and divided the infarction zones as follows: 0 meaning no detected infarction, 1 meaning small/lacunar ischemic lesion, 2 being larger than lacunar but smaller than the whole irrigation zone of the MCA (or basilar artery), and 3 including whole area of the occluded vessel and additional involvement of the anterior or posterior vessels. Non-parametric tests (Fisher exact test, unpaired *t* test, and Mann–Whitney test) were selected to analyze the data, given the sample size and normality of distribution. A median (interquartile range, Q1–Q3) was used for data description. *p* < 0.05 was considered to be statistically significant.

## 3. Results

In the COVID-19 group, there were three active and nine post-COVID-19 patients. The median from COVID-19 infection to AIS was 3 weeks (Q1–Q3, 1–22). One of the patients had a positive history for SARS-CoV-2 infection but the timing was unknown.

Data on baseline laboratory results and prior clinical data are shown in Table 1.

Baseline characteristics of these two groups did not differ in comorbidities such as arterial hypertension, diabetes mellitus, or atrial fibrillation. Although patients from the non-COVID-19 group were older than patients in the COVID-19 group (median 81 vs. 76), it was nonsignificant (*p* = 0.059). On the contrary, the COVID-19 group had a significantly higher percentage of males compared to the non-COVID-19 group (76.9% vs. 40%). The COVID-19 group had significantly higher values of leukocytes, neutrophils, neutrophil/leukocyte ratios, ASL, ALT, LDH and CRP, and lower values of lymphocytes compared to the control group.

Neurological characteristics and therapy-related data are presented in Table 2.

In the COVID-19 group, large vessel occlusion was localized in the anterior circulation in nine patients (69.23%), of which six patients had occlusion in M1 (66.6%), one patient in the M2 segment (11.1%), and two patients had T-occlusion (22.2%). The remaining four patients had occlusion of the basilary artery (30.7%). In the control group, anterior circulation stroke was found in 51 patients (92.7%), as follows: 37 patients had occlusion in M1 segment (67.3%), 5 had occlusion in M2 segment (9%), 9 patients had T-occlusion (16.4%), and only 4 patients had stroke in posterior circulation (7.3%). Comparison of vascular territories where stroke occurred showed that the COVID-19-related group had stroke more often in posterior circulation (30.77% vs. 7.27%), while the control group had stroke more often in anterior circulation (92.73% vs. 69.23%), *p* = 0.039. In the COVID-19 group, there were two (15.4%) wake-up strokes compared to the control group (N = 10, 18.2%). The median NIHSS upon arrival was not different in the non-COVID-19 group compared to the COVID-19 group (median 16 vs. 14, *p* = 0.238). The treatment approach did not differ significantly between groups (*p* = 0.196). The aspiration technique was the most common approach during the procedure; it was used in 69.2% of the COVID-19 patients and in 80% of the control patients. A combination of aspiration and stent retriever was used in 15.4% (COVID-19 group) and in 16.4% (control group). Because of difficult anatomy or other reasons, the procedure was unsuccessful in 15.4% of COVID-19 patients and in 3.6% in the control group. Successful reperfusion (mTICI 2B, 2C and 3) was accomplished in 10/13 COVID-19 patients (76.92%), with mTICI 3 in 8/10 patients (80%), and in 38/55 (69.09%) patients in the control group with the mTICI 3 in 31/38 (81.6%). None of the COVID-19 patients had complications of procedure such as intracerebral hematoma (ICH) or subarachnoid hemorrhage (SAH). In the control group, three (5.4%) patients had SAH and seven (12.7%) had ICH on a control CT performed 24 h after mechanical thrombectomy. Multivariate regression analysis showed that the strongest predictors of a good TICI score (>2B) were antiplatelet therapy (OR 4.67), followed by diabetes (OR 1.96), hypertension (OR 1.45), and COVID-19 (OR 1.28). However, no odd ratio was significant, presumably due to a relatively small number of participants included (Table 3).

Finally, at 90 days follow-up, a good outcome (mRS 0–2) was achieved in 33.33% of COVID-19 patients and in 40% patients in the control group (*p* = 0.751). Multivariate regression analysis showed relatively low and non-significant odds ratios for a good mRS score (Table 4), probably due to the relatively small number of participants included in the study.

One third of patients with prior COVID-19 infection died in the timespan of 3 months after the stroke, which was a similar percent to the patients who died in the control group (28%), (*p* = 0.732). Out of the five COVID-19 patients who died in the timespan of 3 months after AIS, three patients died on the 4th day after the mechanical thrombectomy; one patient had a history of coronary artery disease and had cardiac bypass surgery in the past. He developed changes in the ECG (wide QRS, ST denivelation); another one also underwent cardiac failure and the third was diagnosed with severe urinary infection. The fourth patient died 34 days after suffering acute ischemic stroke under unknown circumstances. The cause of death of the last patient from the COVID-19 group remained unknown, since he did not die in our hospital. Median time from stroke onset to fatal outcome was 4 days (Q1–Q3, 4–26.5).

In the non-COVID-19 group, out of 14 patients who died in the timespan od 3 months after acute ischemic stroke, 5 of them died following an unsuccessful recanalization of the occluded artery. The other nine died of respiratory or cardiac arrest, with a complication of urinary infection in one patient and COVID-19 pneumonia in another. The median time from stroke onset to fatal outcome was 10 days (Q1–Q3, 6–14).

## 4. Discussion

The main result of our center’s experience on mechanical thrombectomy in patients showed that the success of MT was comparable in COVID-19 and non-COVID-19 patients, although some clinical and laboratory characteristics were different between the two groups of patients. The main advantage of this study is that it compared the results of mechanical thrombectomy in COVID-19 patients with a non-infected control group from the same time period during a year of the COVID-19 pandemic. During this time, we performed 13 thrombectomy procedures on patients with emergent large-vessel occlusion that had a positive SARS-CoV-2 test at the time point or history of COVID-19 infection with a median of SARS-CoV-2 detection of 22 days, although some studies reported on a shorter period between the infection and the occurrence of stroke [11,12]. The procedural results during this period were consistent with our pre-COVID-19 institutional experience. While, at the beginning of the COVID-19 pandemic, there were significantly fewer operations and hospital admissions, similar to reports from other centers [13,14,15], the Thrombectomy Management Board at our hospital modified the conductance of AIS patients. The agreement was to continue referring patients for mechanical thrombectomy from all regional hospitals, including those with suspected or proven COVID-19 infection, through the same protocol before the COVID-19 pandemic. Patients with COVID-19 from our center were treated the same as patients negative for COVID-19, and their onset-to-door and door-to-needle times did not significantly differ. Our study showed that the frequency of AIS within the COVID-19 group was higher in men, in contrast to non-infected patients, in whom the proportion of acute ischemia was more frequent in women. Although recent studies confirmed that men and women are equally likely to acquire COVID-19 infection, men have a higher risk of severe illness and worse outcomes [16]. There are several possible explanations for this phenomenon, such as higher expression of angiotensin-converting enzyme-2 (ACE 2) receptors for coronavirus in males, and immunological differences caused by sex hormones [17]. The answer could also lie in different lifestyles because, in many cultures, men have a higher proportion of alcohol and tobacco consumption [18]. Moreover, studies have shown that women were more responsive and had better adherence to epidemiological measures during the COVID-19 pandemic than men [19]. One explanation could be that part of the patients who had a mild clinical presentation and a minor neurological deficit did not come to the hospital at all, or were late for admission due to the fear of being infected with COVID-19. It is probable that the majority of that group was made up of female patients. That being said, it should be noted that there are also conflicting reports by Petik et al. that showed that neurological COVID-19 complications such as stroke did not affect one sex more than the other [20], and by Novikova et al., who showed that predominantly females with COVID-19 developed strokes [21]. Considering our sample size, further and more detailed research is needed to determine which sex is more affected. The median age of COVID-19 patients in our study was 76 years, which is in accordance with several other reports [22]. Moreover, we found no statistical difference between COVID-19 and control non-infected patients regarding age, although some studies report COVID-19 stroke patients being younger [12,23]. The possible explanation for this could be that our patients with COVID-19 infection did not have a severe form of the disease and that the emergency services managed to somewhat adapt to the pandemic, so that patients could present in the hospital within the therapeutic time window. Moreover, a certain percentage of ischemic strokes in patients with severe COVID-19 infection probably remained unrecognized due to prevailing respiratory symptoms, sedation, or poor general condition in the intensive care unit [24]. Our study showed a substantial number of middle cerebral artery occlusion (M1 segment) as a cause of AIS in both groups, which followed the results of some previous studies [9,25]. Interestingly, we observed a significant prevalence of AIS in the vertebrobasilar territory in COVID-19 patients, which is in line with some previous reports [26], although some studies of COVID-19 patients demonstrated a higher proportion of extracranial cerebral vessel dissection as an etiology of ischemic stroke and unexpectedly higher involvement of the anterior cerebral artery [25]. This can be attributed to a complex mechanism of neurovascular pathological changes caused by the infection of COVID-19 that include simultaneous damage to the endothelium, activation of inflammatory mediators, hypercoagulable states, and, as a result, the emergence of atypical, multiple occlusions and dissection [26]. Acute ischemic stroke can also be a manifestation of certain hematological diseases [27], but our investigation of other underlying causes was limited due to the retrospective nature of the study. Since the beginning of the pandemic, multiple studies have established a favored role of an elevated inflammatory response in COVID-19 patients, which was more emphasized in those who were severely affected by the disease and were treated in the intensive care unit. CRP levels, leukocytes and neutrophil levels have been proven to be higher in infected patients, and our laboratory analysis was in accordance with those studies [28,29,30,31,32]. Furthermore, the neutrophil–lymphocyte ratio was higher in our COVID-19 patients. The ratio was found to be a significant factor in establishing the severity of COVID-19 infection, and higher mortality in males [33,34]. Some studies have investigated the role of elevated liver enzymes in correlation with severity of COVID-19 and mortality, which may also burden the patient and elevate the risk of complications such as stroke. Elevated AST and AST/ALT levels correlated with severity of COVID-19 and mortality [35]. Our laboratory analysis was in accordance with these studies. When analyzing the outcome of mechanical thrombectomy procedures, our report showed similar results to some studies regarding a favorable recanalization mTICI score (>2B), which was comparable in COVID-19 and non-COVID-19 groups, as reported earlier [11,36,37]. Studies, including ours, did not find a correlation between procedure-related complications such as sICH or SAH [11,12,36,37], and occurrence of ICH was comparable with large studies [38]. The modified Rankin Scale score, as a rank of functional independence after AIS, showed no difference between our groups, as previously reported [37], although some studies reported higher mortality rate in COVID-19 patients [25,36]. Larger groups of patients should be investigated to establish the correlation between SARS-CoV-2 infection and higher mortality after acute ischemic stroke. In our study, thromboprophylactic therapy was used in a significantly higher percentage in the COVID-19 group compared to the non-COVID-19 group, although that did not affect the occurrence of AIS. A study by Katsoularis et al. showed increased incidence of venous thromboembolisms [39] and some even showed that patients who received anticoagulation therapy after being discharged had lower incidence of thrombotic events [40]. Further studies should investigate which therapy could optimally protect against thromboembolic complications, including stroke, and during which time period after recovering from SARS-CoV-2 infection it should be administered. The main strength of our study is that it was conducted in a single comprehensive stroke center that covers an area of approximately 1 million people. All diagnostic and therapeutic procedures were standardized and carried out by experienced clinicians in stroke management. Two main limitations of our study were its retrospective nature and a small number of patients in the COVID-19 group.

## 5. Conclusions

Although there were differences in some clinical and laboratory parameters between the COVID-19 and non-COVID-19 groups, the outcomes of mechanical thrombectomy were equal.

## Figures and Tables

**Table 1:** Baseline laboratory results and prior clinical characteristics of COVID-19 and control (non-COVID-19) groups.

	COVID-19 Group (N = 13)	Non-COVID-19 (Control) Group (N = 55)	*p* Value
Age (years), median (Q1–Q3)	76 (59.5–82.5)	81 (73–85)	0.059
Male	10 (76.9%)	22 (40%)	0.028
Hypertension N (%)	10 (76.92%)	32 (58.18%)	0.342
Diabetes mellitus N (%)	3 (23.08%)	8 (14.55%)	0.429
Atrial fibrillation N (%)	5 (38.46%)	11 (20%)	0.168
APTT (s)	21.45	22.4	
median (Q1–Q3)	(20.0–26.2)	(21.2–24.5)	0.935
Leukocyte (×10^6^/L)			
median (Q1–Q3)	10 (7.2–11.75)	7.2 (6.3–8.5)	0.011
Neutrophil (×10^6^/L)			
median (Q1–Q3)	76.2 (68.65–86.9)	67 (57.8–75.9)	0.038
Lymphocyte (×10^6^/L)			
median (Q1–Q3)	13.9 (8.25–22.5)	22.5 (15.2–29.8)	0.038
Neutrophil/lymphocyte (N) ratio median (Q1–Q3)	5.48 (3.16–10.27)	2.87 (1.94–4.86)	0.002
AST (IU/L)			
median (Q1–Q3)	32.5 (19–44.5)	23 (19–32)	0.024
ALT (IU/L)			
median (Q1–Q3)	27 (19.5–49.5)	20 (17–27)	0.016
GGT(IU/L)			
median (Q1–Q3)	35 (16.5–93)	27 (16–40)	0.514
LDH (IU/L)			
median (Q1–Q3)	256.5 (168.8–371)	200.5 (168.5–220.8)	0.01
CRP (mg/L)			
median (Q1–Q3)	21.6 (3.25–70.85)	3.25 (1.77–8.57)	0.0004

Abbreviations: APTT—activated partial thromboplastin time, AST—aspartate aminotransferase, ALT—alanine transaminase, GGT—gamma-glutamyl transferase, LDH—lactate dehydrogenase, CRP—C-reactive protein.

**Table 2:** Neurological status and mechanical thrombectomy results of COVID-19 and control (non-COVID-19) groups.

	COVID-19 Group (N = 13)	Non-COVID-19 (Control) Group (N = 55)	p Value
Thrombotic events (N)	1	1	0.089
Antiplatelet therapy (N, %)	8 (61.54%)	14 (25.45%)	0.020
NIHSS at admission (N) median (Q1–Q3)	14 (11–17)	16 (12–18)	0.238
AIS anterior circulation	9 (69.23%)	51 (92.73%)	0.038
AIS posterior circulation	4 (30.77%)	4 (7.27%)	
NIHSS at discharge (N) median (Q1–Q3)	8 (5–14)	8 (4–14)	0.792
TICI category 2B, 2C, 3 (N, %)	10 (76.92%)	38 (69.09%)	0.741
mRS category (0,1,2)	4/12 (33.33%)	20/50 (40%)	0.751
mRS mortality (6)	4/12 (33.33%)	14/50 (28%)	0.732
Control CT small AIS (0,1)	5 (38.4%)	30 (54.5%)	0.45
Control CT large AIS (3)	4 (30.7%)	13 (23.6%)	

Abbreviations: APTT—activated partial thromboplastin time, NIHSS—National Institute of Health Stoke Scale, TICI—Thrombolysis in cerebral infarction, mRS—modified Rankin Scale, CT—computed tomography.

**Table 3:** Multivariate logistic regression analysis of a good TICI score (>2B) predictors.

Variable	OR (95% CI)	*p*
Intercept	0.61 (0.004–82.60)	0.845
Age (years)	0.99 (0.94–1.04)	0.803
Hypertension	1.13 (0.35–3.63)	0.829
Diabetes melitus	0.90 (0.19–3.83)	0.891
Antiplatelet therapy	0.77 (0.18–3.02)	0.711
Atrial fibrillation	0.71 (0.15–3.18)	0.660
Smoking	1.04 (0.04–15.00)	0.971
Door to needle time (min)	1.00 (0.99–1.02)	0.256
COVID-19	0.54 (0.11–2.30)	0.423

AUC = 0.73, Tjur’s R2 = 0.1483, *p* (log likelihood test for overall model) = 0.2249.

**Table 4:** Multivariate logistic regression analysis of a good mRS score (0–2) predictors.

Variable	OR (95% CI)	*p*
Intercept	276.2 (0.99–203,547)	0.066
Age (years)	0.94 (0.88–1.01)	0.118
Hypertension	1.45 (0.41–5.32)	0.559
Diabetes mellitus	1.96 (0.34–17.86)	0.480
Antiplatelet therapy	4.67 (0.84–42.29)	0.110
Atrial fibrillation	0.23 (0.02–1.37)	0.126
Smoking	0.09 (0.003–1.28)	0.090
Door to needle time (min)	0.9904 (0.97–1.00)	0.2506
COVID-19	1.288 (0.23–10.19)	0.7834

AUC = 0.61, Tjur’s R2 = 0.05, *p* (log likelihood test for overall model) = 0.886.

## Data Availability

Not applicable.
